# Timing of kidney biopsy in type 2 diabetic patients: a stepwise approach

**DOI:** 10.1186/s12882-020-01794-w

**Published:** 2020-04-15

**Authors:** Jyh-Tong Hsieh, Fu-Pang Chang, An-Hang Yang, Der-Cherng Tarng, Chih-Yu Yang

**Affiliations:** 1 Division of Nephrology, Department of Medicine, Chiayi Branch, Taichung Veterans General Hospital, Chiayi, Taiwan; 2 Department of Pathology, Taipei Veterans General Hospital, Taipei, Taiwan; 3 School of Medicine, National Yang-Ming University, Taipei, Taiwan; 4 Institute of Clinical Medicine, National Yang-Ming University, Taipei, Taiwan; 5 Division of Nephrology, Department of Medicine, Taipei Veterans General Hospital, Taipei, Taiwan; 6 Center for Intelligent Drug Systems and Smart Bio-devices (IDS2B), Hsinchu, Taiwan; 7 Department and Institute of Physiology, National Yang-Ming University, Taipei, Taiwan; 8 Stem Cell Research Center, National Yang-Ming University, Taipei, Taiwan

**Keywords:** Diabetic nephropathy, Diabetic retinopathy, Hematuria, Kidney biopsy, Non-diabetic renal disease

## Abstract

**Background:**

Diabetic nephropathy (DN) is the most prevalent cause of renal disease in type 2 diabetic patients and is usually diagnosed clinically. A kidney biopsy is considered when non-diabetic renal disease (NDRD) is suspected, such as rapid progression in renal function impairment and severe proteinuria. Still, there is yet no consensus on the timing of kidney biopsy in type 2 diabetic patients. This study aims to identify markers that can help differentiate between DN and NDRD and guide the decision of kidney biopsy.

**Methods:**

We retrospectively reviewed patients with type 2 diabetes who received kidney biopsy from 2008 to 2017 at Taipei Veterans General Hospital. Ophthalmologist consultation and outpatient records, diagnosis of kidney biopsy, laboratory data, and clinical characteristics were collected.

**Results:**

This study enrolled 160 type 2 diabetic patients, among which 120 (75%) had isolated DN and 40 (25%) had NDRD ± DN (26 had isolated NDRD, and 14 had NDRD superimposed on DN). In multivariate logistic regression analysis, DM duration (odds ratio [OR]: 0.907; 95% confidence interval [CI]: 0.842–0.977; *P* = 0.01), diabetic retinopathy (OR: 0.196; 95% CI: 0.061–0.627; *P* = 0.006), and urinary RBC (OR: 1.068; 95% CI: 1.024–1.115; *P* = 0.002) were independent predictors of NDRD. In patients with diabetic retinopathy (*n* = 112, 70%), the presence of proliferative diabetic retinopathy, pan-retinal photocoagulation, and hematuria were factors predicting NDRD; and in patients without diabetic retinopathy (*n* = 48, 30%), short DM duration and hematuria were factors predicting NDRD.

**Conclusions:**

Using diabetic retinopathy, DM duration, and hematuria, we developed a 3-step approach to stratify patients into three categories with the different likelihoods of having NDRD. Then different strategies could be taken accordingly. Our stepwise approach is easy to follow and may serve as an appropriate and useful tool to help clinicians in making decisions of kidney biopsy in type 2 DM patients presenting with kidney diseases.

## Background

Type 2 diabetes mellitus (DM), with its increasing prevalence, is one of the most crucial health problems [1, 2], and diabetic nephropathy (DN) is the leading cause of kidney failure with replacement therapy worldwide [3, 4].

DN is usually diagnosed clinically based on its typical presentations (i.e., a long-standing duration of diabetes, presence of diabetic retinopathy, albuminuria without hematuria, and gradually progressive loss of eGFR), and absence of clinical or laboratory evidence of other kidney diseases [5–7]. A kidney biopsy is not mandatory for diagnosing DN; otherwise, a biopsy should be considered when patients present with atypical features for DN and a non-diabetic renal disease (NDRD) is suspected.

Diabetes-independent factors can cause kidney injury even in patients with DM, resulting in different types of NDRD such as hypertensive nephrosclerosis, atheroembolic disease, genetic kidney disorders, glomerulonephritis, and all kinds of acute kidney injury [8, 9]. Treatment approaches for DN and NDRD may diverge. For instance, IgA nephropathy, focal segmental glomerulosclerosis, membranous glomerulonephritis, and other primary and secondary glomerular diseases usually benefit from personalized treatment with immunosuppressants, other than conservative treatment, such as glycemic control, blood pressure control, and lipid-lowering therapy [10]. Therefore, a kidney biopsy is crucial for NDRD in making an accurate diagnosis and planning appropriate treatment [3]. Compared with DN, patients with NDRD were reported to have a better prognosis in both overall survival and renal survival [11–13]. NDRD may have better outcomes when these conditions are identified early, and specific treatment are predisposed [10]. If clinicians do not suspect the presence of NDRD, a kidney biopsy will not be performed, and etiologies of NDRD will never be identified. As a consequence, patients with NDRD will be treated as DN, and their outcomes will be jeopardized without proper therapy.

The prevalence of NDRD in DM patients varied widely between previous biopsy-based studies, ranging from 3 to 82.9% [10, 14, 15]. Several factors may explain such high histological variability, including selection criteria, indications, and availability of kidney biopsy, as well as on the population investigated [16]. In particular, the criteria used to select patients with diabetes who would benefit from kidney biopsy were different among the studies. Only a small number of studies evaluated research-indicated biopsies, while the vast majority analyzed clinically indicated biopsies [10]. High variability in the prevalence of NDRD also indicated that there were patients who took unnecessary risks to receive a kidney biopsy with their pathology diagnosis turning out to be DN. Besides, there is no formal practice guideline on when to arrange a kidney biopsy for patients with DM [8, 17].

In clinical practice, the value of kidney biopsy in DM patients is to identify NDRD, so proper treatment could be initiated in time. This study aims to explore factors that could help identify the presence of NDRD. We also attempt to propose a practical approach to guide the decision of kidney biopsy in patients with type 2 DM.

## Methods

### Patients and data collection

We retrospectively reviewed patients with type 2 DM who received kidney biopsy at Taipei Veterans General Hospital, a tertiary-care referral center in Taiwan, between 2008 and 2017. Demographic and clinical data were collected from medical records, including age, gender, height, weight, body mass index (BMI), duration of DM before the biopsy, ophthalmic findings (including the presence of diabetic retinopathy, proliferative diabetic retinopathy [PDR] and macular edema [ME]), and treatment received for diabetic retinopathy before biopsy (including focal photocoagulation [PC], pan-retinal photocoagulation [PRP], intravitreal injection [IVI], and vitrectomy [VT]). We defined type 2 DM according to diagnoses of inpatient and outpatient medical records. The duration of DM was also defined as accurately as possible according to the medical record. Laboratory data immediately before the biopsy was recorded, including serum creatinine, estimated glomerular filtration rate (eGFR, using the CKD-EPI formula [18]), level of HbA1c, urinary red blood cell (RBC) and white blood cell (WBC) counts (presented as numbers of cells per high power field [HPF]), and spot urine protein/creatinine ratio (UPCR). Kidney sizes were recorded according to the kidney ultrasonographic report.

### Kidney biopsy and pathology

Indications of kidney biopsy were based on clinical features suggesting renal diseases other than DN, including recent onset of heavy proteinuria or nephrotic syndrome, persistent hematuria with dysmorphic RBCs or RBC casts, and unexplained rapidly progressive renal failure. The decision on kidney biopsy was at the discretion of each attending nephrologist.

The kidney pathology of each patient was examined by two pathologists who were specialists in kidney diseases. The pathologic criteria for DN included diffuse mesangial expansion with the predominance of an increased mesangial matrix, Kimmelstiel–Wilson nodular lesions, hyaline exudative lesions, and glomerular basement membrane thickening. Based on pathological findings, patients were categorized as isolated DN or NDRD ± DN (isolated NDRD or NDRD superimposed on DN).

### Statistical analysis

Binary variables were expressed in counts and percentages. The chi-square test was used for comparisons of categorical variables. Continuous variables were described as mean ± standard deviation for normally distributed data and as median (interquartile range [Q1, Q3]) for non-normally distributed data. Student’s t-test or one-way analysis of variance (ANOVA) was used for normally distributed data analysis, and the Mann-Whitney U test or Kruskal-Wallis test was used for skewed data analysis. Such variables associated with NDRD in univariate analysis with a significance less than 0.10 were retained for multivariate logistic regression analysis. Independent predictors of NDRD were identified by multivariate logistic regression analysis, with results reported as the odds ratio (OR) and 95% confidence interval (CI). Receiver operating characteristic (ROC) curve analyses were performed to determine the best cut-off value of RBC count and duration of DM for predicting the presence of NDRD. All probabilities were two-tailed, and a *P*-value of less than 0.05 was considered to be statistically significant. Based on the status of the identified NDRD predictors, we divided patients into subgroups; patient distribution in subgroups by pathological diagnosis was examined. After subgroup analysis, we proposed a practical approach to guide kidney biopsy in type 2 DM patients. Data were analyzed using Statistical Package for the Social Sciences (SPSS) for Windows, version 22.0 (IBM Corporation, Armonk, NY, USA).

## Results

**Fig. 1:**
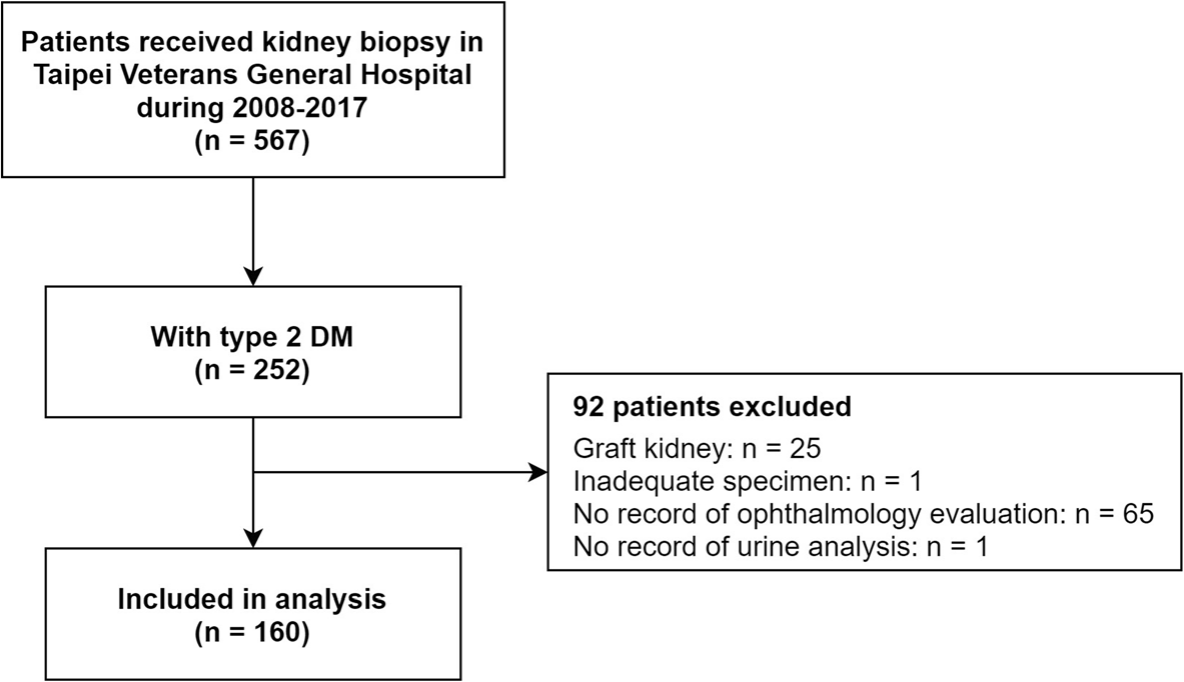
Inclusion and exclusion of our study population

The number of patients with type 2 DM was 252 among the 567 patients who received kidney biopsy. We excluded 92 patients due to the following reasons: biopsy of graft kidney (*n* = 25), an inadequate specimen for pathology report (*n* = 1), lack of ophthalmology evaluation (*n* = 65), and unavailable urinalysis report (*n* = 1). A total of 160 patients was included in the analysis (Fig. 1). All of them received a kidney biopsy for the first time, and there were no known biopsy-proven kidney diseases. Prebiopsy demographics and characteristics are shown in Table 1. Screening for autoimmune diseases (including ANA, ANCA, cryoglobulin …) was performed in some patients but not routinely performed before biopsy in every patient; we didn’t include these examinations for analysis because of too much missing data.

**Table 1:** Clinical and biochemical characteristics of all patients included

**Characteristics**	**Isolated DN** ***n*** **= 120**	**NDRD ± DN** ***n*** **= 40**	***P-*value**
Gender (male)	67 (55.8%)	32 (80%)	0.006
Age (year)	54.8 ± 11.7	64.8 ± 10.6	< 0.001
Body weight (kg)	67.3 (58.7, 75.4)	71.8 (63, 75.6)	0.246
Height (cm)	163 ± 8.5	162.9 ± 8 .2	0.919
BMI (kg/m^2^)	25.1 (22.5, 28.9)	26.1 (23.8, 28.5)	0.259
Duration of diabetes (yr)	10 (4, 16)	4 (1, 7.5)	< 0.001
Diabetic retinopathy	100 (83.3%)	12 (30%)	< 0.001
PDR	56 (46.7%)	2 (5%)	< 0.001
ME	15 (12.5%)	2 (5%)	0.244
PRP	54 (45%)	2 (5%)	< 0.001
PC	13 (10.8%)	0	0.039
IVI	29 (24.2%)	1 (2.5%)	0.002
VT	8 (6.7%)	0	0.202
HbA1c (%)	7.1 (6.1, 8.8)	6.7 (6, 7.5)	0.139
Serum creatinine (mg/dL)	2.8 (1.7, 4.7)	2.3 (1.5, 4.1)	0.256
eGFR (mL/min/1.73 m^2^)	21.1 (11.8, 40)	28.5 (13.4, 46)	0.257
UPCR (1000 mg/g)	9.1 (5.7, 12.3)	7.6 (3.2, 14.8)	0.546
Urinary red blood cells/HPF	4 (1.4, 8)	8 (4, 15.5)	< 0.001
Urinary white blood cells/HPF	1 (1, 4)	1 (1, 4)	0.307
Right kidney size (cm)	11.5 ± 1.1	11.1 ± 1.3	0.056
Left kidney size (cm)	11.5 ± 1.2	11.2 ± 1.2	0.214

Values were presented as median (Q1, Q3), mean ± SD, or n (%)

*DN* diabetic nephropathy, *NDRD* non-diabetic renal disease, *BMI* body mass index, *PDR* proliferative diabetic retinopathy, *ME* macular edema, *PRP* pan-retinal photocoagulation, *PC* focal photocoagulation, *IVI* intravitreal injection, *VT* vitrectomy, *UPCR* urine protein-to-creatinine ratio

Based on the result of kidney biopsy, 120 (75%) patients had isolated DN, 40 (25%) had NDRD ± DN (26 had isolated NDRD, and 14 had NDRD superimposed on DN). Patients with isolated DN had a significantly longer duration of DM (10 [4, 16] vs. 4 [1, 7.5] years, *P* < 0.001), and higher prevalence of diabetic retinopathy (83.3% vs. 30%, *P* < 0.001) than patients with NDRD ± DN. The urinary RBC count was significantly higher in patients with NDRD ± DN than patients with DN (8 [4, 15.5] vs. 4 [1.4, 8] /HPF, *P* < 0.001). Patients with NDRD ± DN also had significantly older age (64.8 ± 10.6 vs. 54.8 ± 11.7 years, *P* < 0.001) and more male gender (80% vs. 55.8%, *P* = 0.006). There was no difference in UPCR, urine WBC count, HbA1c, eGFR, and kidney sizes between two groups (Table 1). Multivariate logistic regression analysis (included gender, age, DM duration, diabetic retinopathy, PDR, urinary RBC, and right kidney size) disclosed that DM duration (odds ratio [OR]: 0.907; 95% confidence interval [CI]: 0.842–0.977; *P* = 0.01), diabetic retinopathy (OR: 0.196; 95% CI: 0.061–0.627; *P* = 0.006), and urinary RBC (OR: 1.068; 95% CI: 1.024–1.115; *P* = 0.002) were independent predictors of NDRD (Table 2).

**Table 2:** Univariate and multivariate logistic regression analysis of significant predictors of NDRD

**Factor**	**Univariate**			**Multivariate**		
	**OR**	**95% CI**	***P*value**	**OR**	**95% CI**	***P*value**
Male gender	3.164	1.346–7.436	0.008^a^	2.257	0.839–7.607	0.099
Age (year)	1.082	1.043–1.123	< 0.001^a^	1.062	0.999–1.130	0.055
Body weight (kg)	1.003	0.978–1.029	0.814			
Height (cm)	0.998	0.955–1.042	0.918			
BMI (kg/m^2^)	1.016	0.938–1.102	0.692			
Duration of diabetes (yr)	0.906	0.854–0.961	0.001^a^	0.907	0.842–0.977	0.01
Diabetic retinopathy	0.086	0.037–0.196	< 0.001^a^	0.196	0.061–0.627	0.006
PDR	0.055	0.013–0.238	< 0.001^a^	0.181	0.024–1.371	0.098
ME	0.352	0.077–1.616	0.179			
PRP	0.058	0.013–0.251	< 0.001			
PC	0		0.999			
IVI	0.075	0.010–0.575	0.013			
VT	0		0.999			
HbA1c (%)	0.849	0.698–1.032	0.101			
Serum creatinine (mg/dL)	0.985	0.859–1.130	0.835			
eGFR (mL/min/1.73 m^2^)	1.008	0.994–1.023	0.265			
UPCR (1000 mg/g)	1.005	0.948–1.066	0.858			
Urinary red blood cells/HPF	1.041	1.012–1.071	0.005^a^	1.068	1.024–1.115	0.002
Urinary white blood cells/HPF	0.998	0.982–1.014	0.769			
Right kidney size (cm)	0.723	0.517–1.012	0.059^a^	1.033	0.611–1.747	0.903
Left kidney size (cm)	0.819	0.598–1.122	0.214			

*OR* odds ratio, *CI* confidence interval, *NDRD* non-diabetic renal disease, *BMI* body mass index, *PDR* proliferative diabetic retinopathy, *ME* macular edema, *PRP* panretinal photocoagulation, *PC* focal photocoagulation, *IVI* intravitreal injection, *VT* vitrectomy, *UPCR* urine protein-to-creatinine ratio

^a^Variables further included in the multivariate logistic regression analysis

**Fig. 2:**
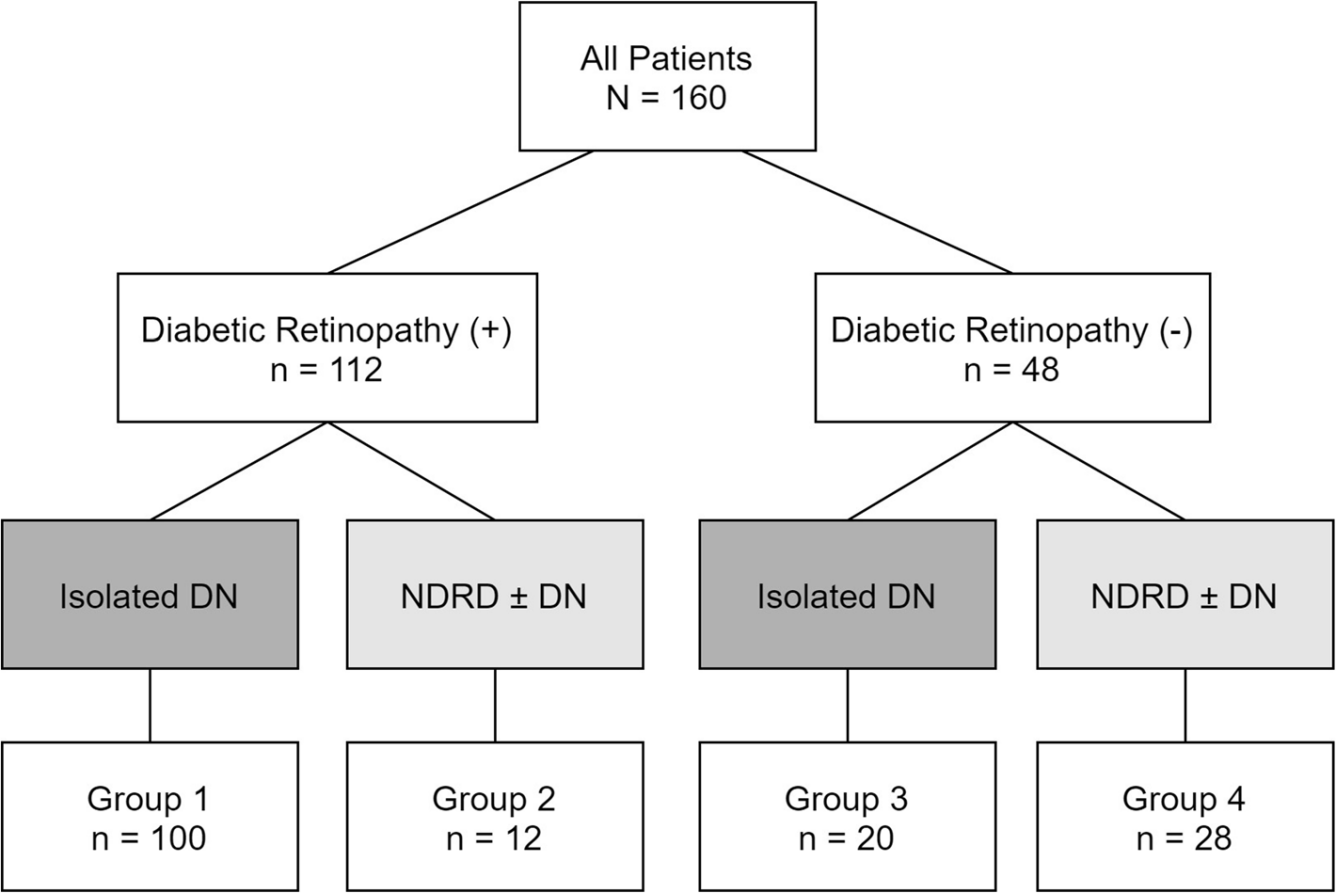
Subgroup analyses in patients with and without diabetic retinopathy. DN, diabetic nephropathy; NDRD, non-diabetic renal disease

We divided all 160 patients into 4 groups according to presence of diabetic retinopathy and diagnosis of kidney pathology (presence of NDRD or not) (Fig. 2). In patients with diabetic retinopathy (Table 3), 100 had isolated DN (group 1) and 12 had NDRD ± DN (group 2); the urinary RBC count was significantly higher in group 2 then group 1 (8 [4, 43] vs. 4 [4, 8] /HPF, *P* = 0.004; ROC cut-off value: 5.5/HPF, AUC = 0.743) (Figure S1), and group1 had a significantly higher prevalence of PDR (56% vs. 16.7%, *P*

**Table 3:** Clinical and biochemical characteristics of patients with diabetic retinopathy

**Characteristics**	**Isolated DN** **(Group 1)** ***n*** **= 100**	**NDRD ± DN** **(Group 2)** ***n*** **= 12**	***P*-value**
Gender (male)	55 (55%)	10 (83.3%)	0.06
Age (year)	55.3 ± 11.5	58.8 ± 8.2	0.111
Body weight (kg)	67.3 (56.3, 77.4)	71.8 (67.4, 74)	0.451
Height (cm)	163.4 ± 8.1	165.3 ± 8.8	0.477
BMI (kg/m^2^)	24.4 (22.2, 28.4)	25.5 (24.2, 28.3)	0.623
Duration of diabetes (yr)	10 (4, 15.8)	6 (3.5, 7.5)	0.217
Diabetic retinopathy			
PDR	56 (56%)	2 (16.7%)	0.01
ME	15 (15%)	2 (16.7%)	1
PRP	54 (54%)	2 (16.7%)	0.029
PC	13 (13%)	0 (0%)	0.354
IVI	29 (29%)	1 (8.3%)	0.176
VT	8 (8%)	0 (0%)	0.596
HbA1c (%)	7.1 (6.3, 9.1)	7.8 (6.5, 9.6)	0.437
Serum creatinine (mg/dL)	2.9 (1.6, 4.7)	3.7 (1.4, 10.8)	0.316
eGFR (mL/min/1.73 m^2^)	20.4 (10.9, 39.9)	19.1 (4.3, 49.7)	0.366
UPCR (1000 mg/g)	9.1 (6.2, 12.5)	7.2 (3.1, 11.9)	0.214
Urinary red blood cells/HPF	4 (4, 8)	8 (4, 43)	0.004
Urinary white blood cells/HPF	1 (1, 4)	1 (1, 4)	0.69
Right kidney size (cm)	11.6 ± 1.1	11.7 ± 1.2	0.785
Left kidney size (cm)	11.5 ± 1.2	11.8 ± 1.0	0.481

Values were presented as median (Q1, Q3), mean ± SD, or n (%)

*DN* diabetic nephropathy, *NDRD* non-diabetic renal disease, *BMI* body mass index, *PDR* proliferative diabetic retinopathy, *ME* macular edema, *PRP* panretinal photocoagulation, *PC* focal photocoagulation, *IVI* intravitreal injection, *VT* vitrectomy, *UPCR* urine protein-to-creatinine ratio

= 0.01) and PRP (54% vs. 16.7%, *P* = 0.029). In patients without diabetic retinopathy (Table 4), 20 had isolated DN (group 3) and 28 had NDRD ± DN (group 4); the urinary RBC count was significantly higher in group 4 than group 3 (6 [4, 15.5] vs. 3.5 [1, 4] /HPF, *P* < 0.001; ROC cut-off value: 6.0/HPF, AUC = 0.786) (Figure S2), and group 3 had significantly longer duration of DM (10 [3.5, 19] vs. 2 [1, 7.5] years, *P* = 0.004; ROC cut-off value: 4.5 years, AUC = 0.745) (Figure S3).

**Table 4:** Clinical and biochemical characteristics of patients without diabetic retinopathy

**Characteristics**	**Isolated DN** **(Group 3)** ***n*** **= 20**	**NDRD ± DN** **(Group 4)** ***n*** **= 28**	***P*-value**
Gender (male)	12 (60%)	22 (78.6%)	0.163
Age (year)	62 ± 10.1	67.3 ± 10.6	0.087
Body weight (kg)	69.6 ± 11.5	69.7 ± 10.6	0.962
Height (cm)	161 ± 10.3	161.9 ± 8	0.742
BMI (kg/m^2^)	26.8 ± 3.5	26.5 ± 3.1	0.741
Duration of diabetes (yr)	10 (3.5, 19)	2 (1, 7.5)	0.004
HbA1c (%)	6.9 (5.7, 8.6)	6.5 (5.9, 7.2)	0.391
Serum creatinine (mg/dL)	2.4 (1.7, 3.4)	2 (1.5, 3)	0.305
eGFR (mL/min/1.73 m^2^)	25.2 (15.3, 41.5)	32.7 (18.8, 46)	0.25
UPCR (1000 mg/g)	7.7 (4.4, 12)	10 (3.4, 16.1)	0.605
Urinary red blood cells/HPF	3.5 (1, 4)	6 (4, 15.5)	< 0.001
Urinary white blood cells/HPF	1 (1, 4)	1 (1, 4)	0.522
Right kidney size (cm)	10.9 ± 0.9	10.8 ± 1.3	0.854
Left kidney size (cm)	11.3 ± 1.1	10.9 ± 1.2	0.291

Values were presented as median (Q1, Q3), mean ± SD, or n (%)

*DN* diabetic nephropathy, *NDRD* non-diabetic renal disease, *BMI* body mass index, *UPCR* urine protein-to-creatinine ratio

**Fig. 3:**
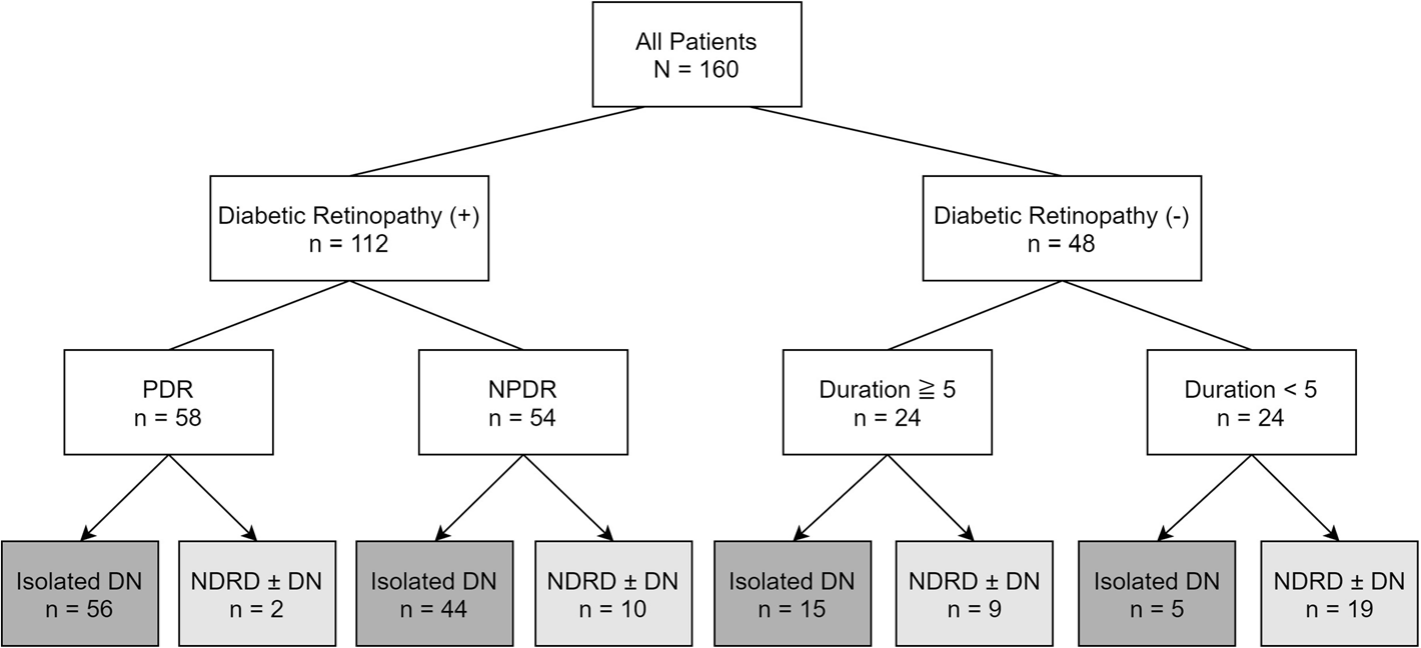
Subgroup analyses: dividing patients according to diabetic retinopathy, proliferative diabetic retinopathy, and duration of diabetes. PDR, proliferative diabetic retinopathy; NPDR, non-proliferative diabetic retinopathy; DN, diabetic nephropathy; NDRD, non-diabetic renal disease

The patients were further divided into eight subgroups based on the presence of diabetic retinopathy, presence of PDR, and DM duration (≥ 5 or < 5 years) (Fig. 3), and subgroup analyses were conducted. In patients with PDR, there was no significant difference between patients with diabetic retinopathy and NDRD ± DN (Table S1). In patients with non-proliferative diabetic retinopathy (NPDR), patients with NDRD ± DN had significantly higher urinary RBC count (8 [4, 43] vs. 4 [4, 8] /HPF, *P* = 0.024) (Table S2). In patients without diabetic retinopathy and DM duration ≥5 years, patients with NDRD ± DN had significantly higher urinary RBC count (8 [4, 32.75] vs. 3 [1, 4] /HPF, *P* < 0.001) and older age at biopsy (73.3 ± 10.4 vs. 62.4 ± 11.0, *P* = 0.025) (Table S3). In patients without diabetic retinopathy and DM duration < 5 years, there was no significant difference between patients with diabetic retinopathy and NDRD ± DN (Table S4). Patient distributions according to the presence of diabetic retinopathy, the presence of PDR, DM duration (≥ 5 or < 5 years), the presence of hematuria (urinary RBC count > 6/HPF), and diagnoses of kidney pathology (isolated DN, NDRD + DN, or isolated NDRD) are shown as Figure S4.

**Fig. 4:**
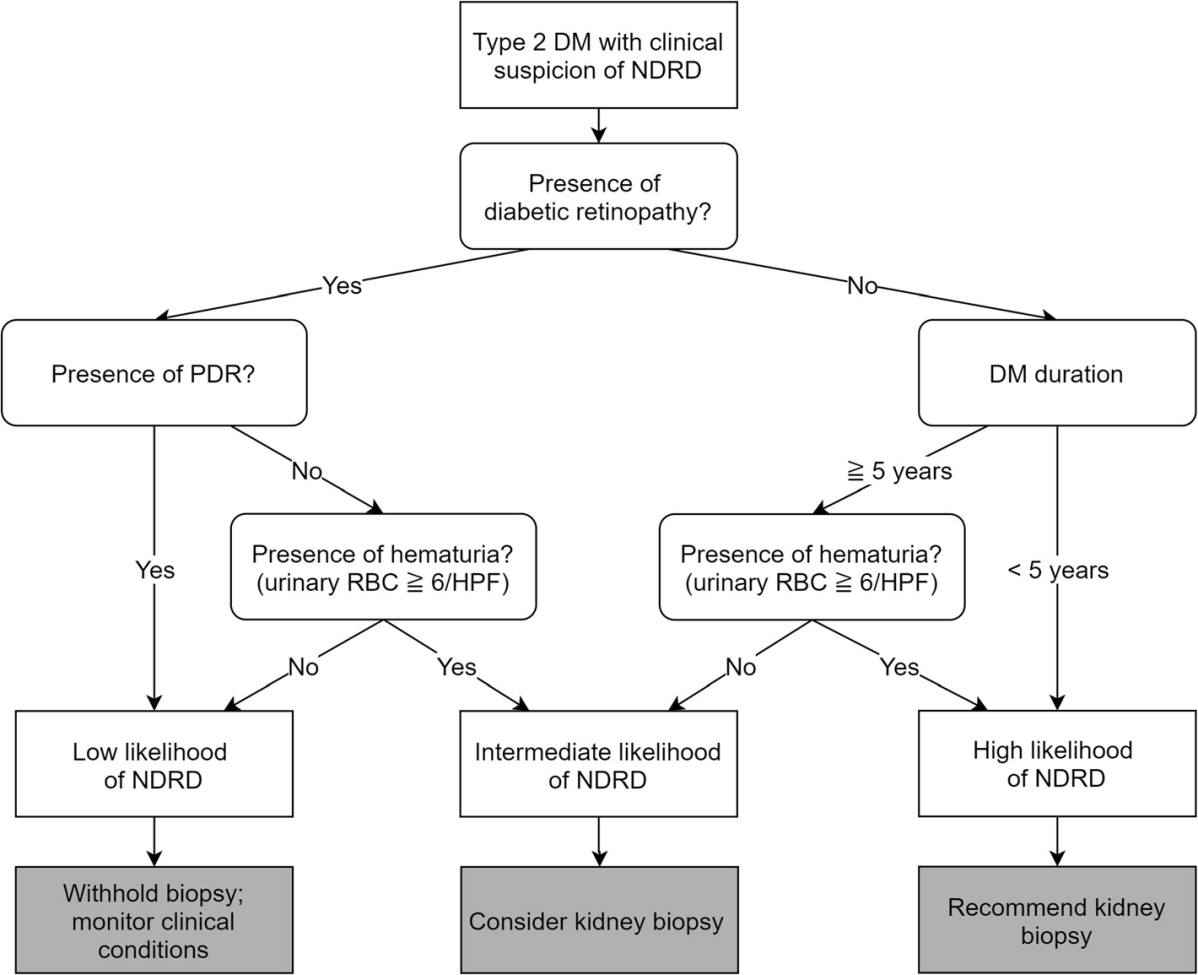
A stepwise approach to guide kidney biopsy in diabetic patients. NDRD, non-diabetic renal disease; PDR, proliferative diabetic retinopathy; RBC, red blood cell

According to the presence of NDRD predictors in the whole population and different subgroups, the patient distribution of this study is shown in Figure S4. Factors we used to divide patients into groups in different steps are listed as follows. Step 1, presence or absence of diabetic retinopathy. Step 2, presence or absence of PDR in patients with diabetic retinopathy and short (< 5 years) or long (≥ 5 years) DM duration in patients without diabetic retinopathy. Step 3, presence (urinary RBC ≥ 6/HPF) and absence (urinary RBC < 6/HPF) of hematuria. By going through the above steps, we divided patients into eight subgroups, and patient numbers of different kidney pathology (isolated DN, NDRD + DN, and isolated NDRD) were listed for each subgroup. The prevalence of NDRD ± DN differed between subgroups and increasing from the left side to the right side in Figure S4. Next, we classified patients into three categories, including low likelihood (2 ~ 10%), intermediate likelihood (17 ~ 28%), and high likelihood (73 ~ 100%) of having an NDRD. Based on the above results, we proposed a 3-step approach to guide kidney biopsy in type 2 DM patients with kidney disease (Fig. 4). The three steps in Fig. 4 are similar to those in Figure S4. Step 3 in Fig. 4 was omitted in the two extreme groups (patients with PDR and patients without diabetic retinopathy & having short DM duration) because their subgroups were similar in terms of the prevalence of NDRD ± DN.

The pathological diagnoses of NDRD identified in this study were listed in Table 5. Within 40 patients with NDRD, the most common diagnoses of NDRD were membranous nephropathy (14, 35%), focal segmental glomerulosclerosis (5, 12.5%), and crescentic glomerulonephritis (5, 12.5%). Membranous nephropathy (11, 42.3%), focal segmental glomerulosclerosis (4, 15.4%), and chronic tubulointerstitial nephritis (3, 11.5%) were the most common diagnoses in the 26 patients with isolated NDRD. Crescentic glomerulonephritis (4, 28.6%), membranous nephropathy (3, 21.4%), and IgA nephropathy (3, 21.4%) were the most common diagnoses in the 14 patients with NDRD superimposed on DN. There was statistically more crescentic glomerulonephritis (*P* = 0.024) and more IgA nephropathy (*P* = 0.014) in the NDRD + DN group than in the isolated NDRD group.

**Table 5:** Pathological diagnosis of NDRD

**Type of NDRD**	**All** **(*n*** **= 40)**	**Isolated NDRD** **(*n*** **= 26)**	**NDRD + DN** **(*n*** **= 14)**	***P*value**
Membranous nephropathy	14 (35%)	11 (42.3%)	3 (21.4%)	0.187
Focal segmental glomerulosclerosis	5 (12.5%)	4 (15.4%)	1 (7.1%)	0.452
Crescentic glomerulonephritis	5 (12.5%)	1 (3.8%)	4 (28.6%)	0.024
IgA nephropathy	3 (7.5%)	0	3 (21.4%)	0.014
Chronic tubulointerstitial nephritis	3 (7.5%)	3 (11.5%)	0	0.186
Minimal change disease	2 (5%)	2 (7.7%)	0	0.287
Lupus nephritis	2 (5%)	1 (3.8%)	1 (7.1%)	0.648
Amyloidosis	1 (2.5%)	1 (3.8%)	0	0.457
Cryoglobulinemic glomerulonephritis	1 (2.5%)	1 (3.8%)	0	0.457
Fibrillary glomerulonephritis	1 (2.5%)	0	1 (7.1%)	0.168
Poststreptococcal glomerulonephritis	1 (2.5%)	0	1 (7.1%)	0.168
Hypertensive nephropathy	1 (2.5%)	1 (3.8%)	0	0.457
Acute interstitial nephritis	1 (2.5%)	1 (3.8%)	0	0.457

*NDRD* non-diabetic renal disease, *DN* diabetic nephropathy

## Discussion

Several pathological diagnoses had been identified in patients with NDRD. Membranous nephropathy and IgA nephropathy were the most common two pathological diagnoses in diabetic patients in the Asian population [10, 19–23]. In our study population, 25% of patients had NDRD (40 out of 160). Membranous nephropathy was the most common finding of NDRD (35%), followed by focal segmental glomerulosclerosis (12.5%), crescentic glomerulonephritis (12.5%), IgA nephropathy (7.5%), and chronic tubulointerstitial nephritis (7.5%). The prevalence of IgA nephropathy was lower than previous reports. The distribution of pathological diagnoses is similar in patients with isolated NDRD, but the most common diagnosis in patients with NDRD superimposed on DN was crescentic glomerulonephritis (28.6%) (Table 5).

We identified a few factors predicting the presence of NDRD, including the absence of diabetic retinopathy, short DM duration, and the presence of hematuria. The definition of hematuria varies in previous studies [16], and the cut-off value for DM duration was not consistent. The presence of diabetic retinopathy is clear-cut (yes or no), and in most circumstances, it is routinely checked when DM patients were present with renal disease. That’s why we chose diabetic retinopathy as the base of patient stratification. The close association between diabetic retinopathy and DN had been well analyzed. Ninety-five percent of patients with type 1 DM and DN also have diabetic retinopathy [17], and diabetic retinopathy is also associated with preclinical histological damage in type 1 DM patients [24]. In patients with type 2 DM, Tone et al. [23] demonstrated that diabetic retinopathy had the highest sensitivity (87%) and specificity (93%) in predicting the presence of DN. According to the KDOQI 2007 guidelines, the cause of CKD is attributable to DN in most people with diabetes if macroalbuminuria or microalbuminuria plus retinopathy is present [25]. However, diabetic retinopathy is concordant with DN in only about 60 to 65% of cases; its absence does not generate a high negative predictive value for the diagnosis of diabetic nephropathy [17]. Another study also suggested that diabetic retinopathy might be a poor predictor of DN, in which DN was present in about 50% of diabetic patients without diabetic retinopathy. In comparison, up to 40% of patients with diabetic retinopathy had other renal diseases [26]. In our study, diabetic retinopathy was associated with isolated DN (*P* < 0.001). In predicting NDRD ± DN, the absence of diabetic retinopathy had sensitivity and specificity as 70 and 83.3%, respectively. All patients with diabetic retinopathy had DN, but 12 (10.7%) of them also had NDRD (NDRD+DN). In patients without diabetic retinopathy, 22 (45.8%) had DN, and 20 (41.7%) of them had isolated DN in kidney pathology. Although there was a strong association between DN and diabetic retinopathy, the present study indicated that diabetic retinopathy alone had an imperfect predictive value [27]. In addition, the decision of biopsy cannot solely rely on the presence and absence of diabetic retinopathy.

We also examined factors reflecting the severity of diabetic retinopathy, including PDR, ME, and specific treatment received (such as PC, PRP, IVI, and VT). To our best knowledge, there were scarce studies analyzed these issues. A meta-analysis that examined four studies suggested that PDR may be a highly specific indicator for DN (pooled sensitivity = 25%; pooled specificity = 98%) [27]. In our study, the presence of PDR, PRP, PC, and IVI was associated with isolated DN (Table 1). In patients with diabetic retinopathy (group 1 vs. group 2; Fig. 2), significant differences were found on PDR and PRP between patients with isolated DN and NDRD ± DN (Table 3). The absence of PDR and absence of PRP had similar sensitivity and specificity (83.3 and 56%, versus 83.3 and 54%) in predicting NDRD ± DN. We can find that the more severe the diabetic retinopathy was, the lower the likelihood of having NDRD. The presence and absence of PDR could provide additional clues on differentiating NDRD ± DN from isolated DN in patients who had diabetic retinopathy.

Duration of DM is closely related to the prevalence of DN in type 1 patients. The prevalence rate of microalbuminuria and macroalbuminuria will increase after 10 years [25]. However, it’s difficult to define the onset of type 2 DM accurately; therefore, the known duration is less strongly related to DN [17, 25]. Several studies still showed that DM duration was shorter in type 2 DM patients with NDRD than patients with DN [19, 20, 28–32]. In our study, patients with NDRD ± DN had a shorter duration of DM than patients with isolated DN (Table 1). In subgroup analyses, the difference could be found in patients without diabetic retinopathy (group 3 vs. group 4; Table 4) but not in patients with diabetic retinopathy (Table 3). The difference in DM duration might come from patients without diabetic retinopathy, and the duration of DM is also a factor predicting the presence of NDRD within this subgroup.

The presence of hematuria has been considered as one of the atypical features suggesting the presence of NDRD in the previous guidelines [25] and reports [16, 28, 30, 32–37]. However, there are also some studies suggesting that hematuria is not an uncommon finding in patients with typical DN (between 35 and 78%) and thus is not useful in predicting NDRD [38–41]. Compared with NDRD, DN is characterized by a different pattern of glomerular lesions, which might result in a different type of hematuria. The most likely mechanism of hematuria in DN might be resulted from areas of aneurysmal dilatation in glomerular capillaries with subsequent rupture and pathological changes in the glomerular basement membrane [42]. Dysmorphic RBCs in the urine sediment may be more useful than microhematuria for indicating NDRD [16, 33]. In our study, patients with NDRD ± DN had significantly more urinary RBC than patients with isolated DN within all patients included and in most of the subgroup analyses. There was no significant difference in urinary RBC in only two subgroups; patients with PDR and patients without diabetic retinopathy and having short DM duration (< 5 years), in which the prevalence of NDRD was lowest and highest, respectively.

We further divided patients with NDRD into glomerulonephritis (*n* = 35) and other NDRD (*n* = 5), there were more urinary RBC in glomerulonephritis than that in isolated DN patients (*P* = 0.003). In addition, hematuria was not different between glomerulonephritis and other NDRD (*P* = 1.000), as well as other NDRD vs. isolated DN (*P* = 0.159) (Table S5). Our results supported the hypothesis that hematuria is an important factor in predicting the presence of NDRD, and it’s also useful in different subgroups, including patients with diabetic retinopathy, patients with NPDR, patients without diabetic retinopathy, and patients without diabetic retinopathy & having long DM duration.

It’s still challenging to distinguish NDRD from patients with DM according to clinical presentation, and there is yet no consensus on detailed criteria to identify patients in whom kidney biopsy is mandatory. Previous studies have demonstrated several clinical manifestations which are predictive of the presence of NDRD, including the absence of diabetic retinopathy, short duration of DM, normal blood sugar, low level of HbA1c, presence of hematuria, heavy proteinuria, and low blood pressure [9, 10, 16, 19, 20, 27–35, 37, 42–51]. However, the above NDRD predictors are actually DN-related, not NDRD-related. Therefore, it’s more appropriate to use these factors in predicting DN rather than predicting NDRD. Besides, the presence of NDRD and the presence of DN should be independent episodes; that is, even if one has factors suggesting a high likelihood of DN, we cannot rule out the possibility of coexisting NDRD. Due to the above conditions, it’s challenging to develop a widely accepted diagnostic tool to tell us which patient should receive kidney biopsy, and there is still no available guideline on this issue.

By using our 3-step approach, patients will be stratified into three categories by the likelihood of having NDRD. In patients with a high likelihood, we recommend performing a kidney biopsy to avoid missed diagnosis of NDRD. In patients with a low probability, a prompt kidney biopsy might increase the unnecessary risk of complications. Under such circumstances, a biopsy could be re-evaluated after a period of time. As for patients with an intermediate likelihood, the decision of biopsy should be judged by the clinical context and experience of each clinician. If a patient doesn’t receive kidney biopsy initially, re-evaluation is indicated either periodically or when clinical condition changes (e.g., increasing urinary RBC count). A kidney biopsy will be suggested once the patient becomes classified into a high likelihood of NDRD in repeated evaluation. Previous studies have identified various predictors of NDRD [19]; correlations with each predictor were presented, but they are not applicable in daily practice. Our stepwise approach provides an easy way to define patients as the different likelihood of having NDRD, and clinicians could take different strategies accordingly. The decision on kidney biopsy or not can be more difficult in some specific scenarios, such as patients with relative contraindications for kidney biopsy. Our stepwise approach can be particularly useful in such conditions and help to make the decision making of kidney biopsy more accurate.

Our study has some limitations. Firstly, this is a biopsy-based study. Kidney biopsies were performed for diagnostic purposes but not for research purposes, and selection bias must exist. All the patients included had been selected by clinicians before biopsy and could not represent the true population of patients with type 2 DM and kidney disease. Besides, the indications of kidney biopsy were not standardized in this retrospective study. Patients presented with typical findings of DN would not receive a biopsy and were therefore excluded from our study. As a single-center study in Taiwan, the results can only be applied in the Asian population. This is a retrospective study with limited population size, and baseline characteristics such as age and gender were not similar between isolated DN group and NDRD ± DN group. For the evaluation of hematuria, the data of dysmorphic RBCs was not available in every patient, so we could not assess the diagnostic value of glomerular hematuria. Our analyses only included data at the time of biopsy. Previous data of renal function, proteinuria, and hematuria were not available, which precluded us from analyzing these variables. Without baseline renal function, we didn’t know whether a patient had acute kidney injury, which is one of the crucial indications of kidney biopsy. The optimal approach for patients with acute kidney injury might be different from other DM patients. Our 3-step approach stratifies patients into 3 categories with varying likelihoods of having NDRD, and there is no standard measure and no standard definition on such risk stratification. Besides, this is a descriptive study that can help hypothesis generation but not to address causal relationships. Further prospective research is warranted to validate this approach.

## Conclusions

In conclusion, a kidney biopsy is essential for diabetic patients to diagnose NDRD accurately, and outcomes will be improved in NDRD patients once timely treatment is applied. However, loose criteria for performing kidney biopsy will yield unnecessary risks of bleeding complications, especially in patients under high risks, such as having high blood pressure and bleeding diathesis. Even though several clinical factors had been identified as predictors of NDRD, but we still don’t know precisely who should receive a kidney and who should not, and there is no detailed consensus or guideline on this issue. We proposed a 3-step approach to stratify diabetic patients into three categories with different likelihoods of NDRD, by using the presence of diabetic retinopathy, DM duration, and hematuria. Our stepwise approach is easy to follow and may serve as a useful tool to help clinicians in making decisions of kidney biopsy in type 2 DM patients presenting with kidney diseases.

## Supplementary information

**Additional file 1:Figure S1.** The receiver operating characteristic (ROC) curve of urinary RBC predicting NDRD in patients with diabetic retinopathy. The ROC area under the curve (AUC) = 0.743.

**Additional file 2:Figure S2.** The receiver operating characteristic (ROC) curve of urinary RBC predicting NDRD in patients without diabetic retinopathy. The ROC area under the curve (AUC) = 0.786.

**Additional file 3:Figure S3.** The receiver operating characteristic (ROC) curve of duration of diabetes predicting NDRD in patients without diabetic retinopathy. The ROC area under the curve (AUC) = 0.745.

**Additional file 4:Figure S4.** Patient distribution according to diabetic retinopathy, proliferative diabetic retinopathy, DM duration (≥ 5 or < 5 years), hematuria (urine RBC count > 6 /HPF), and diagnosis of kidney pathology. The prevalence of the non-diabetic renal disease in each subgroup is presented at the bottom. PDR, proliferative diabetic retinopathy; NPDR, non-proliferative diabetic retinopathy; DN, diabetic nephropathy; NDRD, non-diabetic renal disease.

**Additional file 5:Table S1.** Clinical and biochemical characteristics of patients with proliferative diabetic retinopathy. **Table S2.** Clinical and biochemical characteristics of patients with non-proliferative diabetic retinopathy. **Table S3.** Clinical and biochemical characteristics of patients without diabetic retinopathy & with DM duration ≥5 years. **Table S4.** Clinical and biochemical characteristics of patients without diabetic retinopathy & with DM duration < 5 years **Table S5.** Urinary red blood cells in different pathological diagnosis.

## Data Availability

The datasets used and analyzed during the current study are available from the corresponding author on reasonable request.

## Abbreviations

ANOVA: Analysis of variance
BMI: Body mass index
CI: Confidence interval
DM: Diabetes mellitus
DN: Diabetic nephropathy
IVI: Intravitreal injection
ME: Macular edema
NDRD: Non-diabetic renal disease
PC: Focal photocoagulation
PRP: Pan-retinal photocoagulation
PDR: Proliferative diabetic retinopathy
RBC: Red blood cell
ROC: Receiver operating characteristic
SPSS: Statistical Package for the Social Sciences
UPCR: Spot urine protein/creatinine ratio
VT: Vitrectomy
WBC: White blood cell
