# Supplementary figures and images for: Timing of kidney biopsy in type 2 diabetic patients: a stepwise approach
Source: BMC Nephrol. 2020 Apr 15;21:131. doi: 10.1186/s12882-020-01794-w (PMC7161016; doi:10.1186/s12882-020-01794-w)

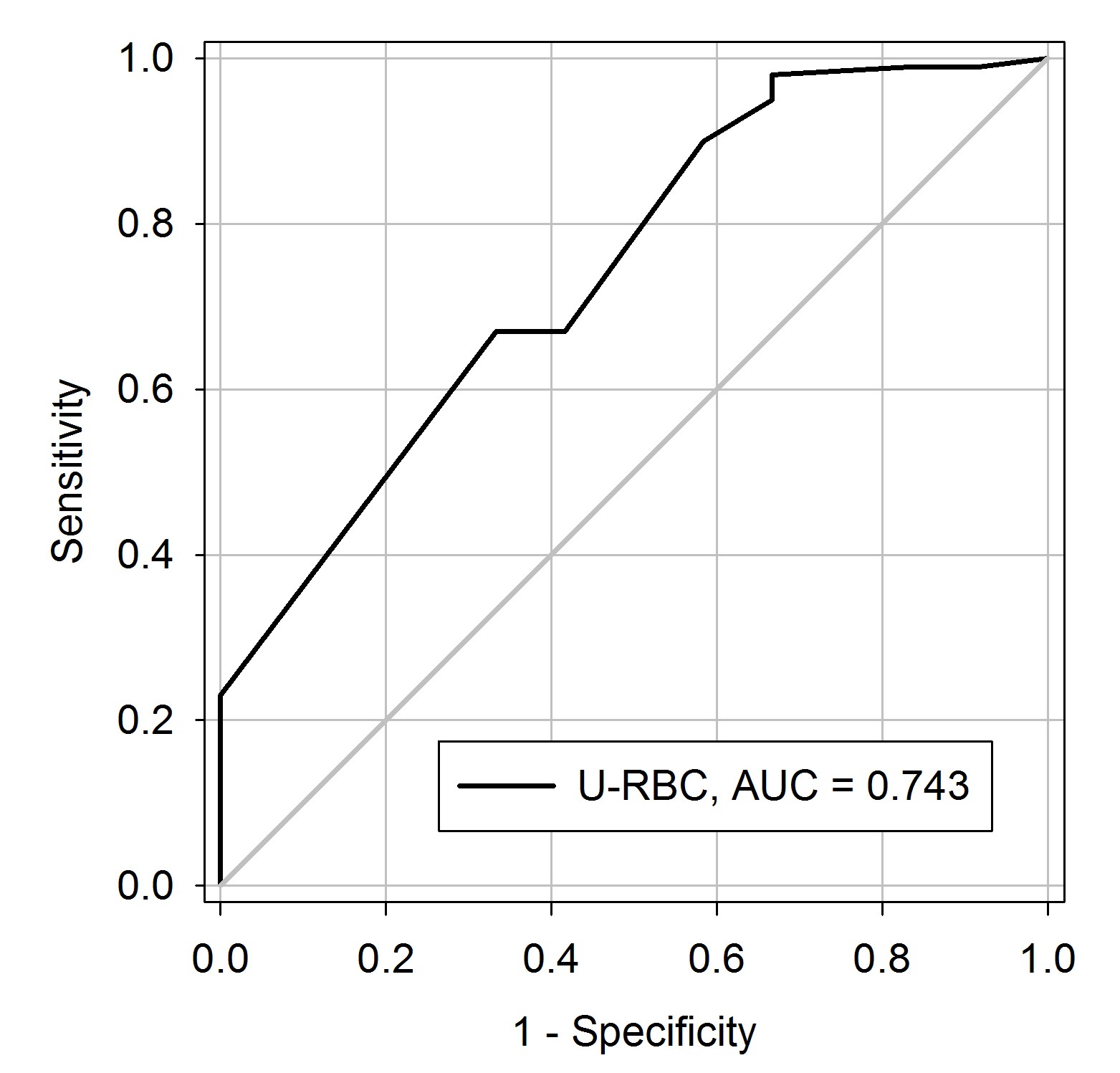

Supplement: Supplementary file 1 — Additional file 1:Figure S1. The receiver operating characteristic (ROC) curve of urinary RBC predicting NDRD in patients with diabetic retinopathy. The ROC area under the curve (AUC) = 0.743. [file 12882_2020_1794_MOESM1_ESM.jpg]

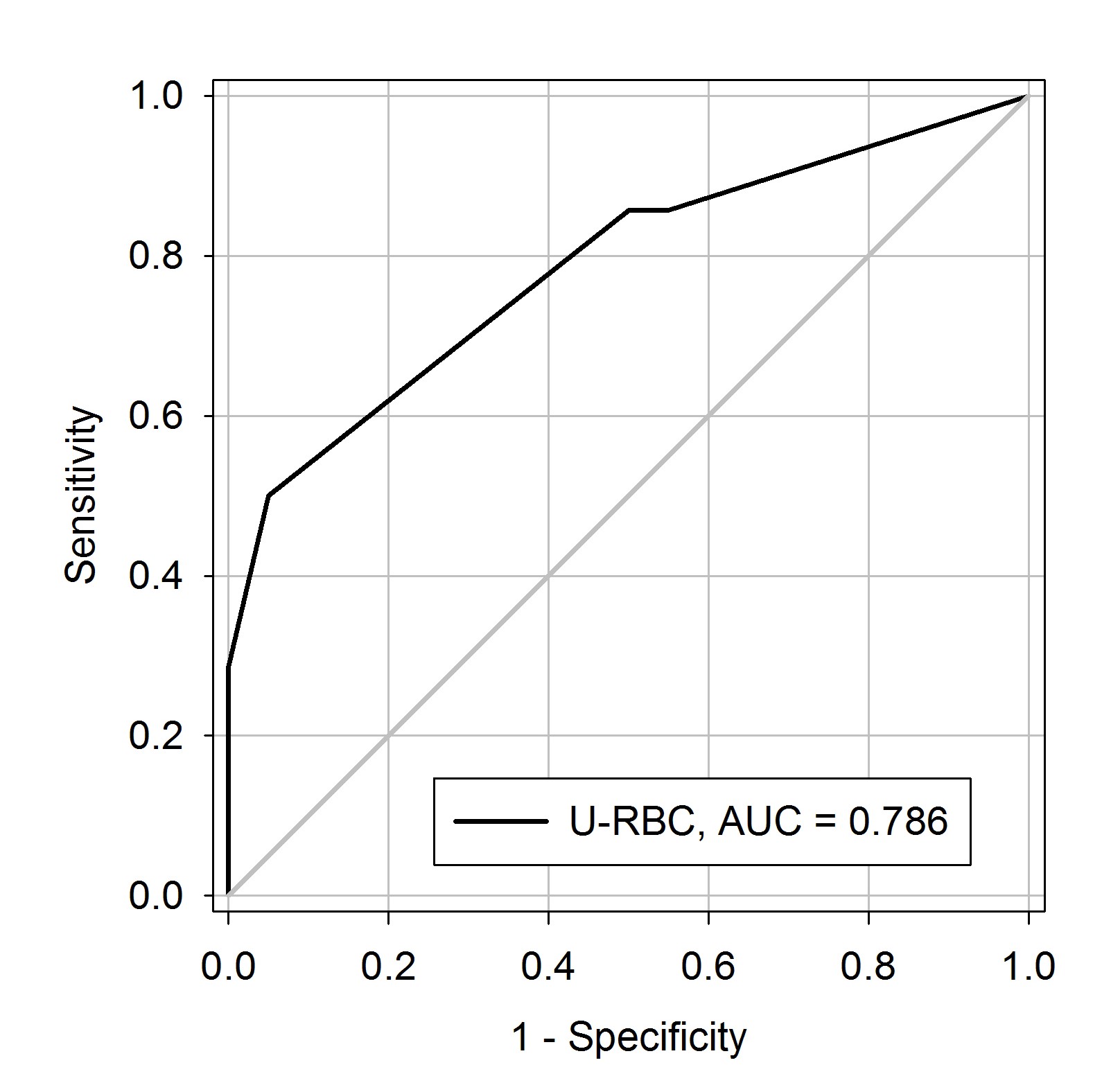

Supplement: Supplementary file 2 — Additional file 2:Figure S2. The receiver operating characteristic (ROC) curve of urinary RBC predicting NDRD in patients without diabetic retinopathy. The ROC area under the curve (AUC) = 0.786. [file 12882_2020_1794_MOESM2_ESM.jpg]

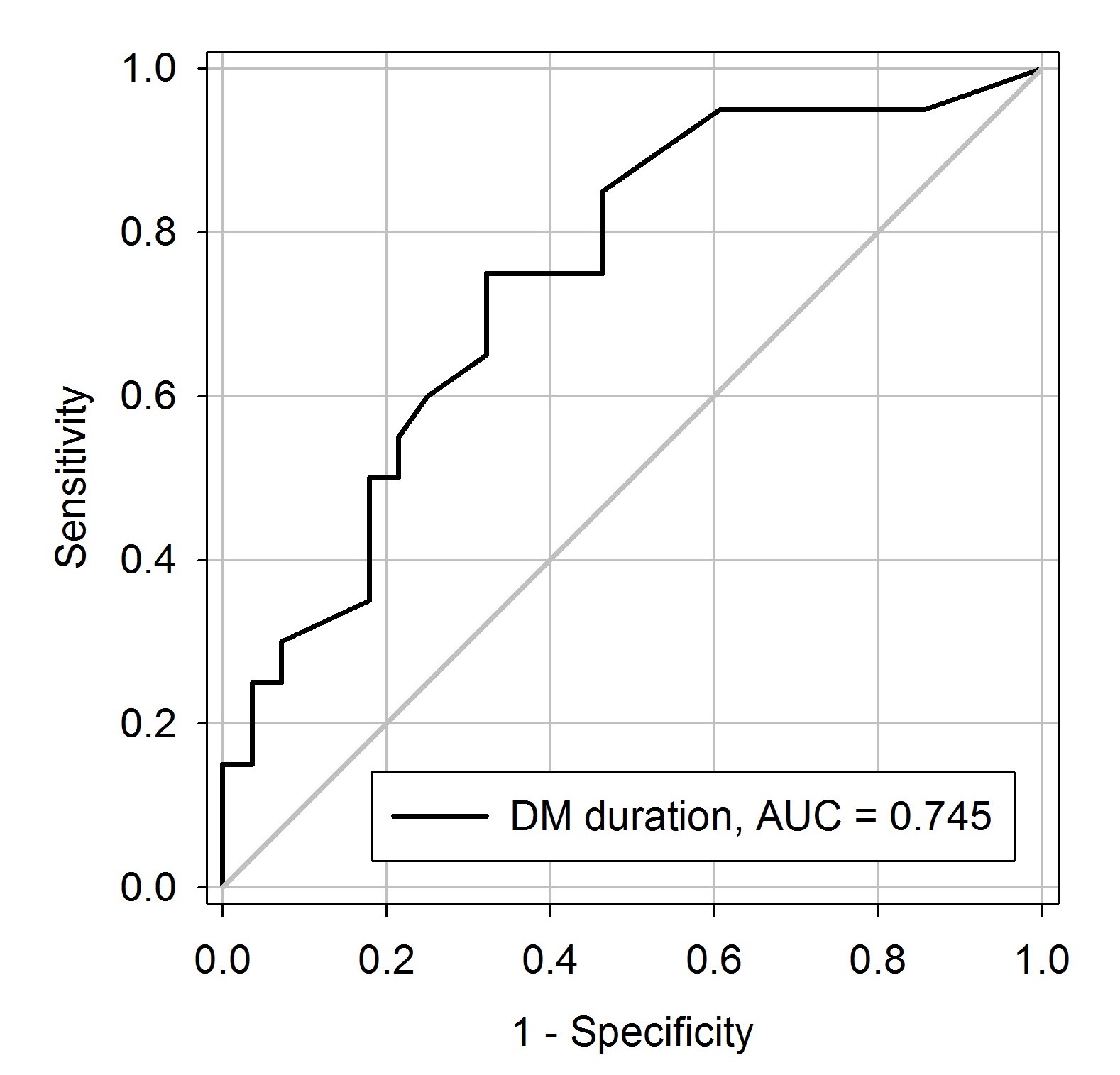

Supplement: Supplementary file 3 — Additional file 3:Figure S3. The receiver operating characteristic (ROC) curve of duration of diabetes predicting NDRD in patients without diabetic retinopathy. The ROC area under the curve (AUC) = 0.745. [file 12882_2020_1794_MOESM3_ESM.jpg]

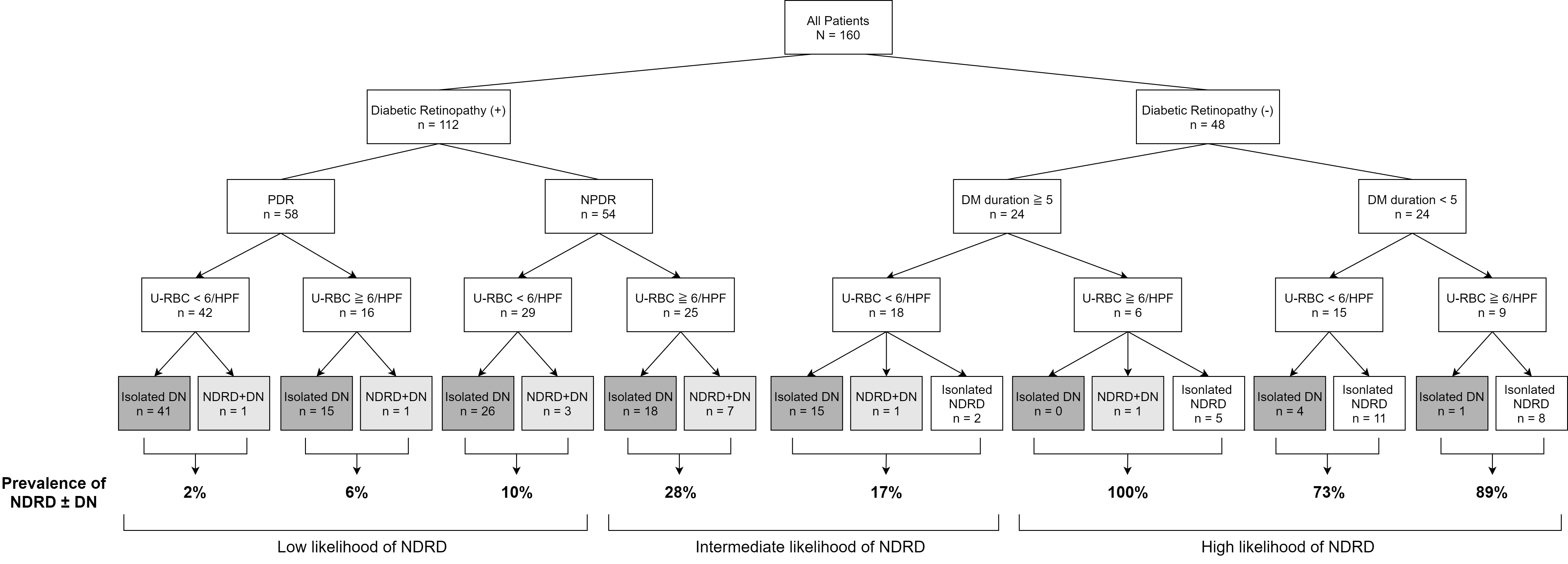

Supplement: Supplementary file 4 — Additional file 4:Figure S4. Patient distribution according to diabetic retinopathy, proliferative diabetic retinopathy, DM duration (≥ 5 or < 5 years), hematuria (urine RBC count > 6 /HPF), and diagnosis of kidney pathology. The prevalence of the non-diabetic renal disease in each subgroup is presented at the bottom. PDR, proliferative diabetic retinopathy; NPDR, non-proliferative diabetic retinopathy; DN, diabetic nephropathy; NDRD, non-diabetic renal disease. [file 12882_2020_1794_MOESM4_ESM.jpg]
