# Supplementary material for: Timing of kidney biopsy in type 2 diabetic patients: a stepwise approach
Source: BMC Nephrol. 2020 Apr 15;21:131. doi: 10.1186/s12882-020-01794-w (PMC7161016; doi:10.1186/s12882-020-01794-w)

**SUPPLEMENTARY MATERIALS**

**Supplementary Tables**

| **Table S1.** Clinical and biochemical characteristics of patients with proliferative diabetic retinopathy. | | | |
| --- | --- | --- | --- |
| **Characteristics** | **Isolated DN n=56** | **NDRD ± DN**  **n=2** | **P-value** |
| Gender (male) | 29 (51.8%) | 1 (50%) | 1 |
| Age (year) | 48.4 ± 10.2 | 52.0 ± 5.7 | 0.628 |
| Body weight (kg) | 70.7 ± 16.8 | 73.5 ± 0.8 | 0.822 |
| Height (cm) | 164.3 ± 8.2 | 157.1 ± 1.6 | 0.224 |
| BMI (kg/m^2^) | 26.0 ± 4.9 | 29.8 ± 0.3 | 0.285 |
| Duration of diabetes (yr) | 10.5 (4, 15) | 5.5 (5, -) | 0.55 |
| Diabetic retinopathy |  |  |  |
| ME | 10 (17.9%) | 1 (50%) | 0.346 |
| PRP | 43 (78.2%) | 2 (100%) | 1 |
| PC | 7 (12.5%) | 0 | 1 |
| IVI | 26 (46.6%) | 1 (50%) | 1 |
| VT | 8 (14.3%) | 0 | 1 |
| HbA1c (%) | 7.1 (6.2, 9.7) | 5.95 (5.9, -) | 0.087 |
| Serum creatinine (mg/dl) | 2.93 (1.82, 4.71) | 8.0 (7.2, -) | 0.05 |
| eGFR (mL/min/1.73 m^2^) | 20.6 (11.4, 36.8) | 6.4 (4.5, -) | 0.05 |
| UPCR (1000 mg/g) | 8.8 (6.5, 15.0) | 11.8 (7.4, -) | 0.691 |
| Urinary red blood cells/HPF | 4 (4, 8) | 27 (4, -) | 0.232 |
| Urinary white blood cells/HPF | 1 (1, 4) | 1 (1, 1) | 0.294 |
| Right kidney size (cm) | 11.7 ± 1.0 | 11.3 ± 1.6 | 0.569 |
| Left kidney size (cm) | 11.8 ± 1.0 | 12.1 ± 1.5 | 0.697 |
| *DN, diabetic nephropathy; NDRD, non-diabetic renal disease; BMI, body mass index; PDR, proliferative diabetic retinopathy; ME, macular edema; PRP, pan-retinal photocoagulation; PC, focal photocoagulation; IVI, intravitreal injection; VT, vitrectomy; UPCR, urine protein-to-creatinine ratio.* | | | |
| *Values were presented as median (Q1, Q3), mean ± SD, or n (%).* | | |  |

| **Table S2.** Clinical and biochemical characteristics of patients with non-proliferative diabetic retinopathy. | | | |
| --- | --- | --- | --- |
| **Characteristics** | **Isolated DN (n=44)** | **NDRD ± DN**  **(n=10)** | **P-value** |
| Gender (male) | 26 (59.1%) | 9 (90%) | 0.08 |
| Age (year) | 59.5 ± 10 | 60.2 ± 8.2 | 0.848 |
| Duration of diabetes (yr) | 10 (4, 17.8) | 6 (3, 11) | 0.233 |
| Diabetic retinopathy |  |  |  |
| ME | 5 (11.4%) | 1 (10%) | 1 |
| PRP | 11 (25%) | 0 | 0.101 |
| PC | 6 (13.6%) | 0 | 0.58 |
| IVI | 3 (6.8%) | 0 | 1 |
| VT | 0 | 0 |  |
| Urinary red blood cells/HPF | 4 (4, 8) | 8 (4, 43) | 0.024 |
| Urinary white blood cells/HPF | 1 (1, 4) | 1 (1, 5) | 0.798 |
| UPCR (1000 mg/g) | 9.4 (5.4, 12) | 6.2 (3, 9.8) | 0.182 |
| HbA1c (%) | 7 (6.3, 8.7) | 8.2 (7.1, 10.3) | 0.076 |
| Serum creatinine (mg/dl) | 2.8 (1.6, 4.7) | 2.5 (1.1, 11.8) | 0.713 |
| Right kidney size (cm) | 11.4 ± 1.1 | 11.8 ± 1.1 | 0.385 |
| Left kidney size (cm) | 11.1 ± 1.3 | 11.7 ± 1 | 0.234 |
| Body weight (kg) | 65.4 (56, 72.1) | 69.5 (60.9, 76.4) | 0.341 |
| Height (cm) | 162.2 ± 7.8 | 167.1 ± 8.7 | 0.104 |
| BMI (kg/m^2^) | 24.2 (21.5, 27.5) | 24.6 (22.8, 26.6) | 0.772 |
| eGFR (mL/min/1.73 m^2^) | 19.1 (10.9, 40.7) | 26.9 (4.2, 60.5) | 0.876 |
| *DN, diabetic nephropathy; NDRD, non-diabetic renal disease; BMI, body mass index; ME, macular edema; PRP, pan-retinal photocoagulation; PC, focal photocoagulation; IVI, intravitreal injection; VT, vitrectomy; UPCR, urine protein-to-creatinine ratio.* | | | |
| *Values were presented as median (Q1, Q3), mean ± SD, or n (%).* | | |  |

| **Table S3.** Clinical and biochemical characteristics of patients without diabetic retinopathy & with DM duration ≥ 5 years. | | | |
| --- | --- | --- | --- |
| **Characteristics** | **Isolated DN n=15** | **NDRD ± DN**  **n=9** | **P-value** |
| Gender (male) | 8 (53.3%) | 8 (88.9%) | 0.178 |
| Age (year) | 62.4 ± 11.0 | 73.3 ± 10.4 | 0.025 |
| Body weight (kg) | 67 (63.2, 71) | 62 (56.6, 69.5) | 0.078 |
| Height (cm) | 162.0 ± 9.7 | 159.8 ± 7.7 | 0.571 |
| BMI (kg/m^2^) | 26.8 ± 3.7 | 24.3 ± 3.1 | 0.115 |
| Duration of diabetes (yr) | 15.1 ± 9.2 | 12.8 ± 5.5 | 0.495 |
| HbA1c (%) | 7.1 ± 1.3 | 6.4 ± 0.7 | 0.089 |
| Serum creatinine (mg/dl) | 2.6 (1.5, 3.5) | 2.4 (1.5, 3.7) | 0.811 |
| eGFR (mL/min/1.73 m^2^) | 22.5 (15.2, 46) | 27 (15.9, 39.2) | 0.743 |
| UPCR (1000 mg/g) | 6.5 (4.4, 11.4) | 4.1 (2.1, 11.7) | 0.313 |
| Urinary red blood cells/HPF | 3 (1, 4) | 8 (4, 32.75) | < 0.001 |
| Urinary white blood cells/HPF | 3 (1, 5) | 4.1 (2.1, 11.7) | 0.108 |
| Right kidney size (cm) | 11.0 ± 1.0 | 10.2 ± 1.3 | 0.144 |
| Left kidney size (cm) | 11.2 ± 1.1 | 10.4 ± 1.4 | 0.161 |
| *DN, diabetic nephropathy; NDRD, non-diabetic renal disease; BMI, body mass index; UPCR, urine protein-to-creatinine ratio.* | | | |
| *Values were presented as median (Q1, Q3), mean ± SD, or n (%).* | | |  |

| **Table S4.** Clinical and biochemical characteristics of patients without diabetic retinopathy & with DM duration < 5 years | | | |
| --- | --- | --- | --- |
| **Characteristics** | **Isolated DN n=5** | **NDRD ± DN**  **n=19** | **P-value** |
| Gender (male) | 4 (80%) | 14 (73.7%) | 1 |
| Age (year) | 62 (54, 67) | 64 (59, 67) | 0.454 |
| Body weight (kg) | 66.8 ± 5.8 | 73.2 ± 9.2 | 0.158 |
| Height (cm) | 158.2 ± 12.4 | 162.9 ± 8.1 | 0.313 |
| BMI (kg/m^2^) | 26.9 ± 3.3 | 27.5 ± 2.6 | 0.638 |
| Duration of diabetes (yr) | 2 (1, 3) | 1 (1, 2) | 0.443 |
| HbA1c (%) | 7.1 ± 2.1 | 6.6 ± 0.8 | 0.63 |
| Serum creatinine (mg/dl) | 2.1 (1.8, 6.6) | 1.9 (1.3, 2.8) | 0.477 |
| eGFR (mL/min/1.73 m^2^) | 33.1 (17.4, 35.5) | 35.5 (18.7, 61.2) | 0.374 |
| UPCR (1000 mg/g) | 12.3 ± 6.8 | 12.2 ± 9.2 | 0.985 |
| Urinary red blood cells/HPF | 4 (1, 6) | 4 (4, 8) | 0.252 |
| Urinary white blood cells/HPF | 1 (1, 1) | 1 (1, 4) | 0.119 |
| Right kidney size (cm) | 10.6 0.8 | 11.1 ± 1.2 | 0.432 |
| Left kidney size (cm) | 11.6 ± 1.1 | 11.2 ± 1.1 | 0.449 |
| *DN, diabetic nephropathy; NDRD, non-diabetic renal disease; BMI, body mass index; UPCR, urine protein-to-creatinine ratio.* | | | |
| *Values were presented as median (Q1, Q3), mean ± SD, or n (%).* | | |  |

| **Table S5.** Urinary red blood cells in different pathological diagnosis | | |  |  |
| --- | --- | --- | --- | --- |
| **Characteristics** | **Glomerulonephritis n=35** | **Other NDRD  n=5** | **Isolated DN n=120** | ***P* value** |
| Urinary red blood cells/HPF | 8 (4, 15.5) | 8 (4, 32.75) | 4 (1.38, 8) | 0.003 |
| *DN, diabetic nephropathy; NDRD, non-diabetic renal disease.* | | |  |  |
| *Values were shown as median (Q1, Q3).* | |  |  |  |

**Supplementary Figures**

**Figure S1.** The receiver operating characteristic (ROC) curve of urinary RBC predicting NDRD in patients with diabetic retinopathy. The ROC area under the curve (AUC) = 0.743.


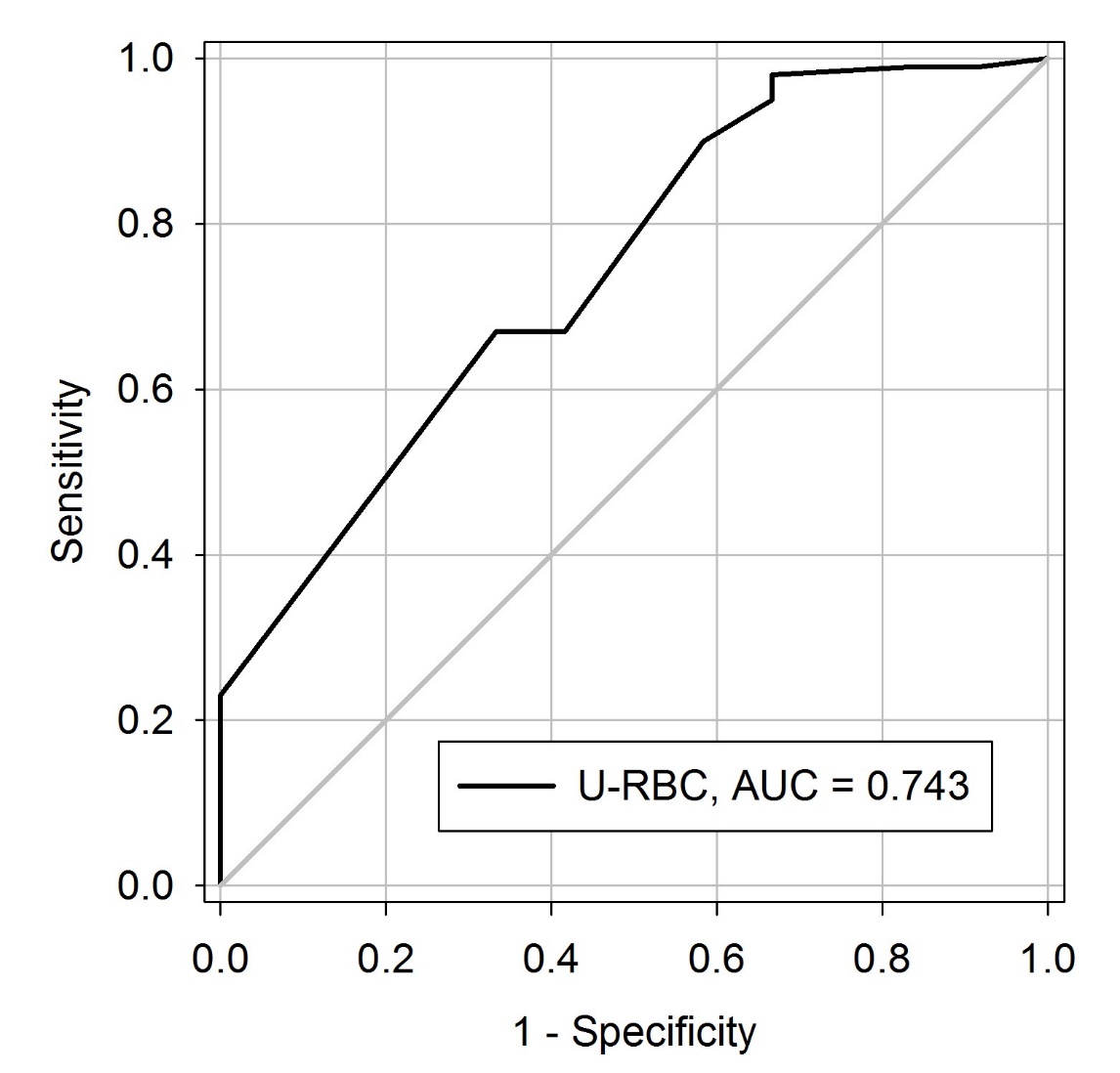


**Figure S2.** The receiver operating characteristic (ROC) curve of urinary RBC predicting NDRD in patients without diabetic retinopathy. The ROC area under the curve (AUC) = 0.786.


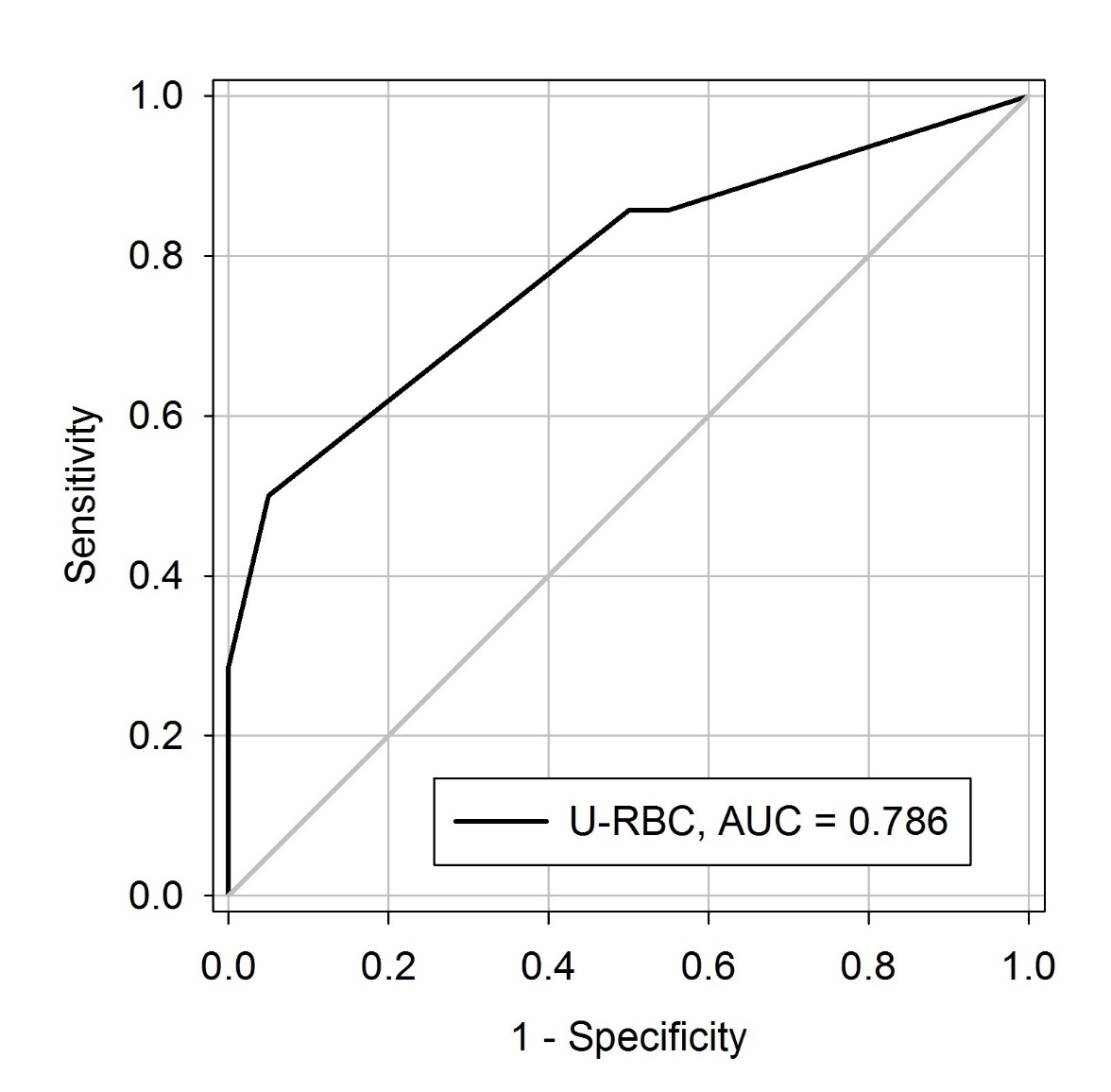


**Figure S3.** The receiver operating characteristic (ROC) curve of duration of diabetes predicting NDRD in patients without diabetic retinopathy. The ROC area under the curve (AUC) = 0.745.


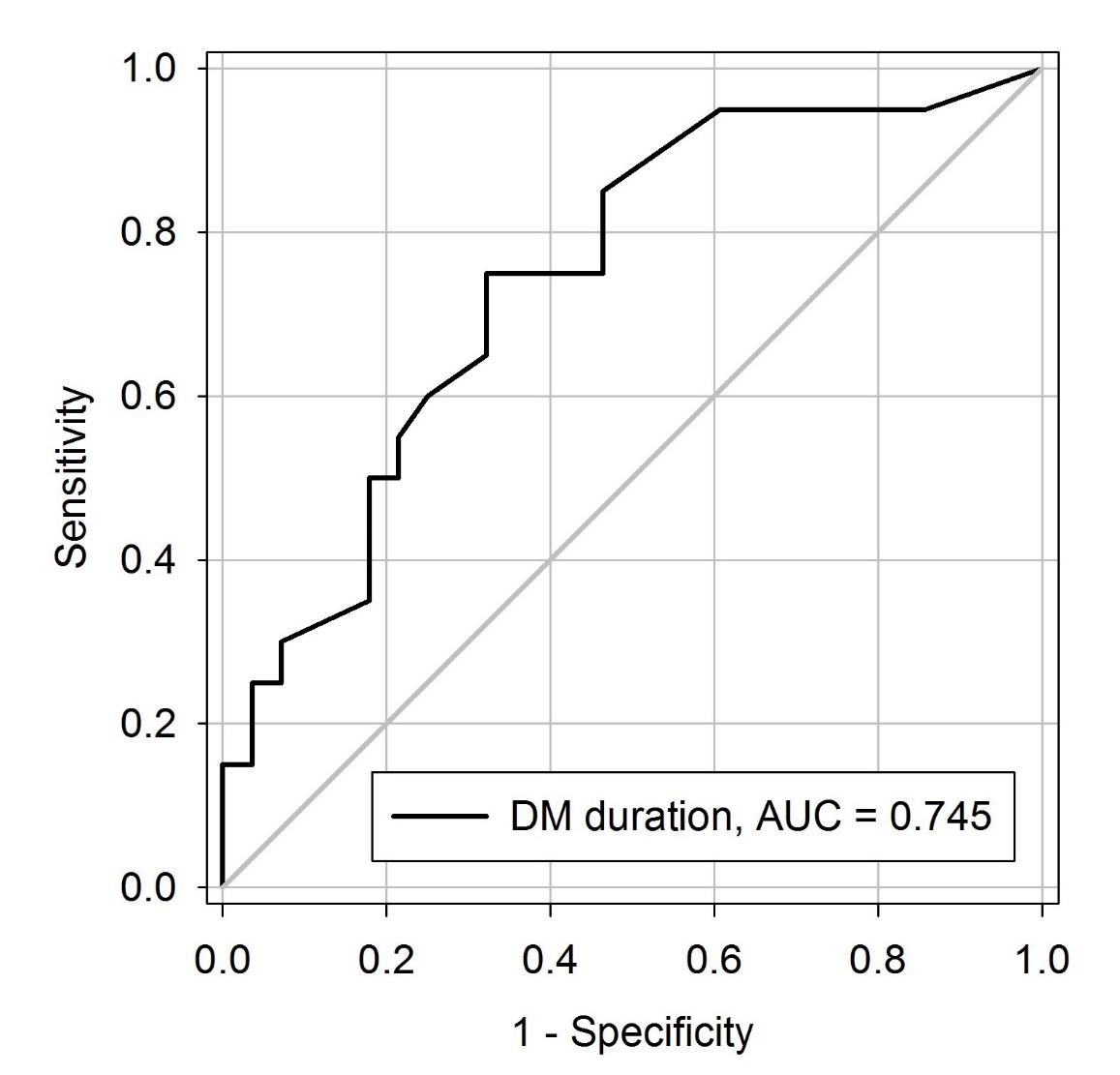


**Figure S4.** Patient distribution according to diabetic retinopathy, proliferative diabetic retinopathy, DM duration (≥ 5 or < 5 years), hematuria (urine RBC count > 6 /HPF), and diagnosis of kidney pathology. The prevalence of the non-diabetic renal disease in each subgroup is presented at the bottom. PDR, proliferative diabetic retinopathy; NPDR, non-proliferative diabetic retinopathy; DN, diabetic nephropathy; NDRD, non-diabetic renal disease.


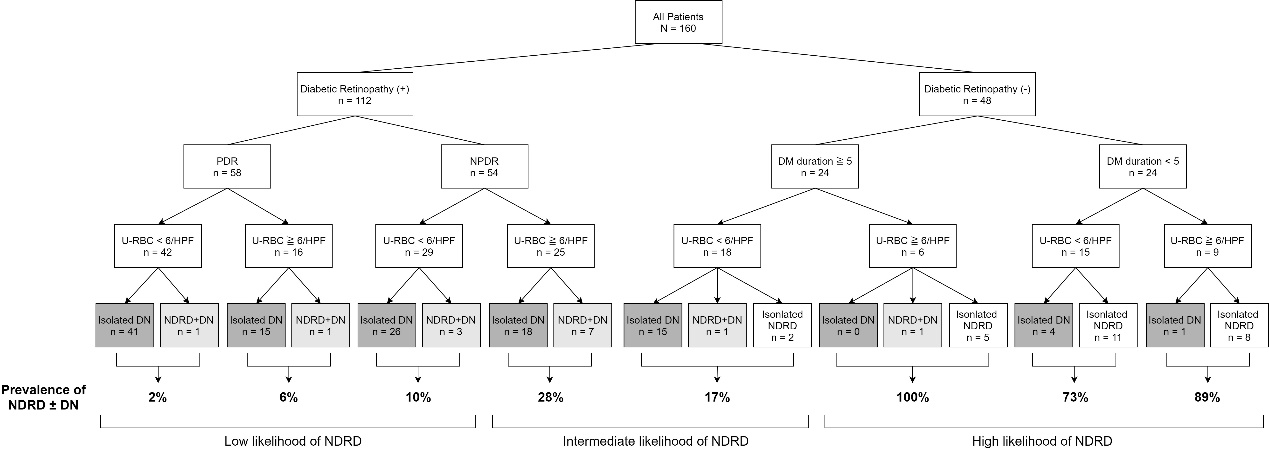

Supplement: Supplementary file 5 — Additional file 5:Table S1. Clinical and biochemical characteristics of patients with proliferative diabetic retinopathy. Table S2. Clinical and biochemical characteristics of patients with non-proliferative diabetic retinopathy. Table S3. Clinical and biochemical characteristics of patients without diabetic retinopathy & with DM duration ≥5 years. Table S4. Clinical and biochemical characteristics of patients without diabetic retinopathy & with DM duration < 5 years Table S5. Urinary red blood cells in different pathological diagnosis. [file 12882_2020_1794_MOESM5_ESM.docx]
